# Differential Myf5 and Myf6 expression and muscle fiber traits in Angora, Hair, Honamlı, and Kilis goats

**DOI:** 10.1007/s11250-025-04277-y

**Published:** 2025-01-27

**Authors:** Büşra Bayrak, Uğur Şen, Dilek Gökçek, Emre Şirin

**Affiliations:** 1 Department of Agricultural Biotechnology, Faculty of Agriculture, Ondokuz Mayis University, 55139 Samsun, Türkiye; 2 Department of Agricultural Biotechnology, Faculty of Agriculture, Kırşehir Ahi Evran University, 40100 Kirsehir, Türkiye

**Keywords:** Indigenous breeds, Goat, Gene expression, Meat production, Kid

## Abstract

The present study was conducted on specific skeletal muscles of six weaned male kids from each of the Angora, Hair, Honamlı, and Kilis goat breeds. The relationships between the expression of myogenic factor 5 (Myf5) and myogenic factor 6 (Myf6) genes and muscle fibre characteristics were analysed. Muscle samples from the longissimus dorsi (LD) and semitendinosus (ST) were collected from six 90-day-old weaned male kids of each breed. Muscle fiber characteristics were assessed through histochemical staining, while expression levels of Myf5 and Myf6 genes were quantified using real-time PCR. Total RNA content in the LD and ST muscles was significantly higher (*p* < 0.05) in Honamlı kids compared to those of the other breeds. Similarly, the expression of Myf5 gene in Honamlı kids was significantly higher (*p* < 0.05) than that observed in kids from the other breeds in LD muscle. Conversely, in the ST muscle, Hair kids exhibited a significantly lower expression of Myf5 (*p* < 0.05) when compared to both Honamlı and Kilis kids Additionally, Kilis kids demonstrated a significant reduced expression of Myf6 gene (*p* < 0.05) relative to the other breeds. The highest expression levels of the Myf6 gene (*p* < 0.05) were detected in the ST muscle of Honamlı and Angora kids, significantly surpassing those observed in Hair and Kilis kids. Moreover, significant correlations (*p* < 0.05) were observed among Myf5 and Myf6 gene expression levels and various muscle fiber characteristics differing across breeds. The results of this study underscore the pivotal role of these myogenic regulatory factors in muscle development, offering insights into the molecular mechanism driving breed-specific muscle growth. This association between gene expression and muscle phenotype could have profound implications for targeted breeding programs aimed at optimizing muscle traits in livestock species.

## Introduction

Goat and kid meat constitute a pivotal component of red meat production in Türkiye. Among the indigenous goat breeds, Angora, Hair, Honamlı, and Kilis are particularly prominent, collectively representing approximately 92% of Türkiye’s goat population (Sen et al. 2020). In Türkiye, goat breeding is predominantly characterized by an extensive system that heavily relies on natural pasture conditions, with concentrated feed rarely utilized (Yilmaz et al. 2012; Sen et al. 2020). The limited application of kid fattening practices within the country has resulted in a significant lack of robust data concerning the fattening potential of indigenous Turkish goat breeds. Therefore, comprehensive research is essential to systematically assess the growth performance and meat yield potential of these breeds under controlled fattening conditions.

An increase in muscle fibers’ size (hypertrophy) and number (hyperplasia) causes the muscle to grow and develop (Siqin et al. 2017). Nutritional level and exercise intensity may affect skeletal muscle mass development or meat quality (Reimers et al. 2014; Sen et al. 2016; Siqin et al. 2017). The development of skeletal muscle mass and meat quality is significantly influenced by both nutritional status and exercise intensity (Reimers et al. 2014; Sen et al. 2016; Siqin et al. 2017). Furthermore, the composition of skeletal muscle fibers plays a crucial role in determining the muscle’s metabolic properties, contraction sensitivity, and postnatal meat quality attributes (Lim et al. 2015; Sen et al. 2016). During the fetal period, the number of muscle fibers is established; however, subsequent differentiation in fiber diameter and composition occurs during the postnatal phase without any alteration in fiber quantity (Du et al. 2010). Despite this understanding, the genomic mechanisms that underlie fiber type differentiation and transformation remain inadequately elucidated (Siqin et al. 2017). Comprehensive investigation into these processes is imperative for advancing our knowledge of muscle biology and enhancing meat quality in livestock species.

Growth rates and carcass yield of livestock are economically important characteristics. Myogenic regulatory factors gene families (MRFs) (Zhong et al. 2013), satellite cells (Bunprajun et al. 2012), and myostatin (Shibata et al. 2006) regulate the growth and development of skeletal muscles. Moreover, MRFs regulate myogenesis from the fetal stage of muscle fiber proliferation, formation, and development to postnatal muscle fiber maturation, differentiation, growth, and function (Zhong et al. 2013; Siqin et al. 2017). In addition, MRFs have an essential role in determining the expression of many different genes and hormones (growth hormone and its receptor, IGF-I, myostatin; MSTN, myosin heavy chain; MyHC isoforms) associated with economic characteristics such as growth, development, fattening performance, and meat yield (Huang et al. 2016). Myogenic factor 5 (Myf5) is considered the first expressed MRFs gene and is regulated by a 140 kb enhancer complex in its regulatory region (Carvajal et al. 2008). Braun and Arnold (1995) reported that Myf5 is responsible for developing and increasing muscle fibers in the embryonic and fetal periods, and myogenic factor 6 (Myf6) is responsible for postnatal muscle maturation and development.

Although previous studies have reported that Hair, Angora, Honamlı, and Kilis indigenous breeds exhibit different phenotypic values in fattening performance, the primary mechanism of this difference has not been revealed; it was based on breed characteristics or environmental factors (Aktaş et al. 2015; Akbaş and Saatçi 2016; Gürsoy et al. 2011; Erol and Ünal 2021). Our previous studies have thoroughly characterized the carcass and meat quality traits, muscle fiber composition, and cellular properties of Angora, Hair, Honamlı, and Kilis kids (Sen et al. 2020, 2021a, b; Sirin 2018). However, to our knowledge, there exists a significant gap in the literature regarding the expression profiles of the Myf5 and Myf6 genes in the kids of these four indigenous goat breeds. Moreover, the potential relationship between the expression profiles of Myf5 and Myf6 and the characteristics of muscle fibers has yet to be determined in these kids. The investigation of Myf5 and Myf6 gene expression, given their critical roles in skeletal muscle development, may provide essential insights into the meat production capabilities of Turkish indigenous goat breeds. Therefore, this study aims to systematically analyze the expression profiles of Myf5 and Myf6 and their correlations with muscle fiber type composition and dimensions in selected skeletal muscles, specifically the longissimus dorsi (LD) and semitendinosus (ST), of 90-day-old male kids at weaning age from the Angora, Hair, Honamlı, and Kilis goat breeds.

## Material and methods

The current study utilized longissimus dorsi (LD) and semitendinosus (ST) muscle samples, stored at −80 °C, obtained from male kids of four indigenous goat breeds: Angora (*n* = 6), Hair (*n* = 6), Honamlı (*n* = 6), and Kilis (*n* = 6), all at 90 days of weaning age. To control for potential confounding factors related to nutrition and exercise on postnatal muscle fiber development and the expression of Myf5 and Myf6 genes, all kids were raised under standardized management and nutritional conditions until weaning. Comprehensive details regarding the rearing practices of the kids and the procedures for muscle sample collection are elaborated upon in the materials and methods section of our preceding publication (Sen et al. 2020).

## Isolation of total RNA and RT-qPCR

Total RNA was isolated from LD and ST muscle samples using a commercial RNA (PureLink ™, RNA Mini Kit, Invitrogen™, 12183018A) isolation kit with the TRIzol Reagent (Thermo Fisher Scientific, USA) as suggested by the manufacturer. Isolated RNase-free DNase was used to remove genomic DNA residue in isolated RNA. Total RNA quantity and quality were examined using a Nanodrop spectrophotometer (Thermo Fisher Scientific, USA) at 260 nm, and RNA integrity was checked by agarose gel electrophoresis 1% (w/v). The isolated RNA samples were diluted to 1 µg/µl, and cDNA synthesis was performed by a commercial kit (BIO-RAD iScript cDNA, 1708890) with reverse transcriptase and designed primers. Obtained cDNA samples were stored at − 20 °C until analyses of qRT-PCR.

Mfy5 and Mfy6 gene expression levels were examined by RT-qPCR method. Primers were designed using online tools (https://www.ncbi.nlm.nih.gov/tools/primerblast/; accessed on 18 March 2023) for the amplification of genes based on the related gene sequences of caprine (Table 1). Glyceraldehyde-3-phosphate dehydrogenase (GAPDH) was used as reference gene to normalize expression of target gene. Primers were purchased from Sentebiolab Company (Ankara, Türkiye).

**Table 1:** Primer sequences for the mRNA expression analysis of genes

Genes	Primer sequence (5′−3′)	Product size (bp)	GenBank
Myf5	F: CACGACCAACCCTAACCAGAG R: TCTCCACCTGTTCCCTTAGCA	129	JF829004
Myf6	F: CGGAGCGCCATTAACTACAT R: AAATCCGCACCCTCAAGATT	113	NM_001285602
GAPDH	F: GCAAGTTCCACGGCACAG R: TCAGCACCAGCATCACCC	79	AF035421

Relative quantification of target genes was performed by qRT-PCR using the CFX96 Touch Real-Time PCR Detection System (Bio-Rad Laboratories, Hercules, CA, USA). qRT-PCR were run with EvaGreen mastermix (5 × HOT FIREPol EvaGreen qPCR Mix Plus, Solis BioDyne, Tartu, Estonya). Total volume of reaction mix was presented in Table 2. Amplification of qRT-PCR was conducted as defined by Huang et al (2016). 2 − ΔΔCt method was used for calculated relative mRNA expression levels of target the genes (Huang et al. 2016).

**Table 2:** The components of real-time PCR reaction mix

Components	Volume (μL)
cDNA	2
10 μmol/L Forward PCR primer	0.5
10 μmol/L Reverse PCR primer	0.5
5 × HOT FIREPol mix	5
DEPC treated water	2
Total volume of reaction mix	10

## Statistical analyses

The analysis of Myf5 and Myf6 gene expression profiles was conducted using a completely randomized design, facilitated by SPSS software (17.0; licensed by Ondokuz Mayıs University). To evaluate significant differences between means, Duncan’s multiple range test was used, with a significance level set at *p* < 0.05. Additionally, Pearson correlation analysis was used to calculate the correlation coefficients between the expression profiles of Myf5 and Myf6 and the composition of muscle fiber types (Type I, IIA, IIB) as well as cross-sectional area. The data for the Pearson correlation analysis were sourced from previous studies conducted by one of the authors (Sirin 2018), which focused on muscle fiber types in the specified muscles.

## Results

**Fig. 1:**
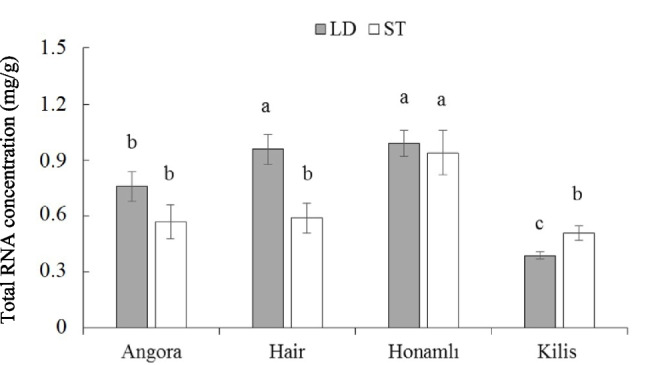
Total RNA concentrations in longissimus-dorsi (LD) and semitendinosus (ST) muscles of weaned kids born from Angora, Hair, Honamlı, and Kilis goat breeds. The error bars represent the standard error of the mean and bars with different letters are significantly different at *p* < 0.05

The total RNA content in LD and ST muscles of weaned male kids from each of Angora, Hair, Honamlı, and Kilis goats are presented in Fig. 1. The total RNA amount in LD muscle was highest in Honamlı and Hair kids compared to Angora and Kilis counterparts. Conversely, Honamlı kids displayed a reduced total RNA content in the ST muscle compared to the other breeds (*p* < 0.05). Furthermore, significant differences in total RNA levels were observed between the LD and ST muscles among Angora, Hair, and Kilis kids (*p* < 0.05), indicating notable variations in muscle-specific gene expression potential across these breeds.

**Fig. 2:**
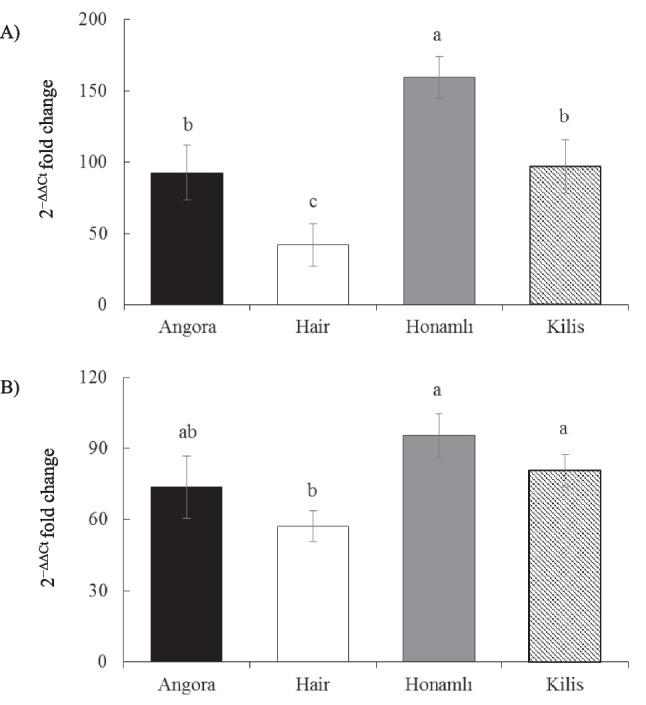
Myf5 gene expressions in longissimus-dorsi (**A**) and semitendinosus (**B**) muscles of weaned kids born from Angora, Hair, Honamlı, and Kilis goat breeds. The error bars represent the standard error of the mean and bars with different letters are significantly different at *p* < 0.05

Myf5 gene expression levels in LD and ST muscles of weaned male kids, each of Angora, Hair, Honamlı, and Kilis Turkish indigenous breeds of goats are presented in Fig. 2. The study showed statistically significant differences between breeds regarding the Myf5 gene expression level in the LD and ST muscles. In the LD muscle, Myf5 gene expression was significantly highest in Honamlı kids (*p* < 0.05), but lowest Hair kids (*p* < 0.05). Notably, Hair kids had diminished Myf5 expression compared to both Honamlı and Kilis kids, except when contrasted with Angora kids in the ST muscle.

**Fig. 3:**
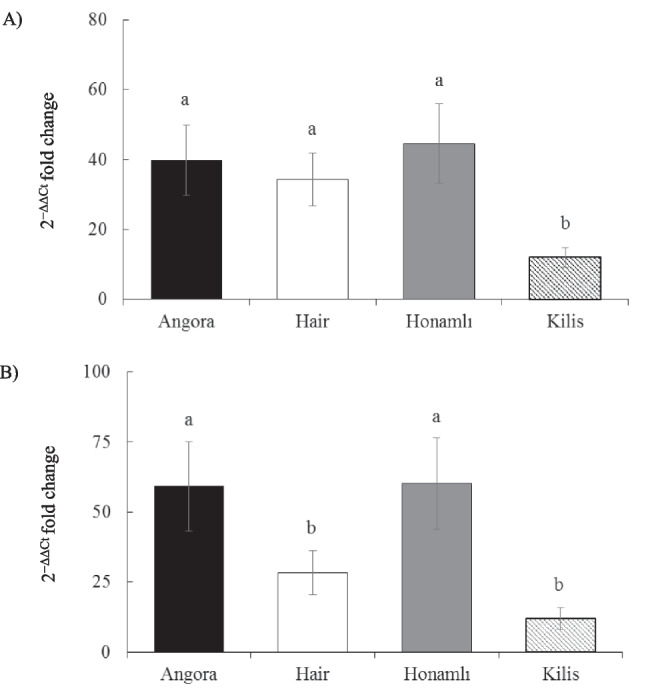
Myf6 gene expressions in longissimus-dorsi (**A**) and semitendinosus (**B**) muscles of weaned kids born from Angora, Hair, Honamlı, and Kilis goat breeds. The error bars represent the standard error of the mean and bars with different letters are significantly different at *p* < 0.05

Myf6 gene expression levels in LD and ST muscles of weaned male kids, each of Angora, Hair, Honamlı, and Kilis Turkish indigenous breeds of goats are presented in Fig. 3. In the current study, there were statistically significant differences between breeds regarding the expression level of the Myf6 gene in the LD and ST muscles. Kilis kids had lower (*p* < 0.05) Myf6 gene expression levels than those of the Honamlı, Hair, and Angora breeds in LD muscle. In the ST muscle, Myf6 gene expression was significantly highest in Honamlı and Angora kids (*p* < 0.05), whereas Hair and Kilis kids demonstrated the lowest expression levels (*p* < 0.05).

Correlation coefficients among the muscle fiber number, cross-sectional area (CSA), Myf5 and Myf6 genes expression in LD and ST muscles of male kids, each of Angora, Hair, Honamlı, and Kilis Turkish indigenous breeds of goats are presented in Tables 3, 4, 5, and 6, respectively. The Myf6 gene expression was significantly negative correlated with the type IIA, IIB, and total fibers number, but significantly positive correlated with the CSA of type I, IIA, IIB fibers as well as average CSA of fibers (*p* < 0.05) in the ST muscles of Angora kids (Table 3). The Myf6 gene expression was significantly negative correlated (*p* < 0.05) with the type I fiber number, but significantly positive correlated (*p* < 0.01) with average and type I muscle fiber CSA in the LD muscle of Hair kids (Table 4). While there was a positive correlation between Myf5 gene expression and the number of type I fibers (*p* < 0.05), a negative correlation was observed between Myf5 gene expressions and type I muscle fibers CSA (*p* < 0.05) in the ST muscle of Hair kids (Table 4). Contrast to gene expression level of Myf6 was significantly negative correlated with number fiber numbers (*p* < 0.05), but significantly positive correlated (*p* < 0.05) with CSA of type IIA fiber. In the ST muscle of Honamlı kids (Table 5), a series of significant positive correlations were observed among Myf6 gene expression, type I (*p* < 0.01), type IIA (*p* < 0.05), and total fiber number (*p* < 0.01). Furthermore, there was a negative correlation (*p* < 0.05) between Myf5 gene expression and type IIA fiber number in the LD muscle of Kilis kids (Table 6). However, a positive correlation between Myf5 gene expression and CSA of type IIA fiber was calculated in the LD muscle of Kilis kids (*p* < 0.01). Additionally, while a negative correlation was obtained between Myf5 gene expression and type I fiber number (*p* < 0.01), a positive correlation was calculated between Myf5 gene expression and CSA of type I fiber in the ST muscle of Kilis kids (Table 6).

**Table 3:** Correlation coefficients among the muscle fiber number, CSA, Myf5 and Myf6 gene expression in Angora kids

Measurements	Gene expression (LD)		Gene expression (ST)	
	Myf5	Myf6	Myf5	Myf6
Type I	−0,419	−0,316	−0,319	−0,435
Type IIA	−0,334	0,129	0,251	−0,677*
Type IIB	0,116	0,521	0,287	−0,896**
Total Fiber	−0,334	0,035	0,207	−0,828**
Type I CSA	0,506	0,173	0,367	0,709*
Type IIA CSA	0,232	−0,393	−0,294	0,954**
Type IIB CSA	−0,180	−0,500	−0,221	0,997**
Average CSA	0,591	0,092	0,032	0,936**

*LD* longissimus-dorsi, *ST* semitendinosus, *CSA* cross-sectional area

^*^p < 0.05, **p < 0.01

**Table 4:** Correlation coefficients among the muscle fiber number, CSA, Myf5 and Myf6 gene expression in Hair kids

Measurements	Gene expression (LD)		Gene expression (ST)	
	Myf5	Myf6	Myf5	Myf6
Type I	−0,180	−0,781*	0,273	0,731*
Type IIA	−0,352	−0,230	−0,513	−0,627*
Type IIB	−0,505	0,361	0,363	−0,362
Total Fiber	−0,426	−0,245	−0,466	−0,526
Type I CSA	0,149	0,947**	−0,378	−0,665*
Type IIA CSA	0,159	−0,130	0,297	0,795*
Type IIB CSA	0,517	−0,398	−0,291	0,349
Average CSA	0,306	0,878**	−0,545	−0,556

*LD* longissimus-dorsi, *ST* semitendinosus, *CSA* cross-sectional area

^*^p < 0.05, **p < 0.01

**Table 5:** Correlation coefficients among the muscle fiber number, CSA, Myf5 and Myf6 gene expression in Honamlı kids

Measurements	Gene expression (LD)		Gene expression (ST)	
	Myf5	Myf6	Myf5	Myf6
Type I	−0,237	0,249	−0,498	0,927**
Type IIA	0,465	0,304	−0,221	0,764*
Type IIB	0,397	0,187	0,456	0,323
Total Fiber	0,347	0,291	−0,261	0,973**
Type I CSA	−0,316	−0,185	0,384	−0,360
Type IIA CSA	−0,476	−0,159	0,064	−0,241
Type IIB CSA	−0,428	−0,284	−0,262	0,162
Average CSA	−0,359	−0,185	0,186	−0,21

*LD* longissimus-dorsi, *ST* semitendinosus, *CSA* cross-sectional area

^*^p < 0.05, **p < 0.01

**Table 6:** Correlation coefficients among the muscle fiber number, CSA, Myf5 and Myf6 gene expression in Kilis kids

Measurements	Gene expression (LD)		Gene expression (ST)	
	Myf5	Myf6	Myf5	Myf6
Type I	−0,038	−0,232	−0,850**	0,014
Type IIA	−0,679*	−0,005	0,076	0,278
Type IIB	−0,390	−0,116	0,169	−0,251
Total Fiber	−0,498	−0,082	0,022	0,240
Type I CSA	−0,096	−0,044	0,935**	0,139
Type IIA CSA	0,876**	−0,278	−0,147	−0,269
Type IIB CSA	0,305	−0,032	−0,235	0,315
Average CSA	0,539	−0,197	0,371	−0,131

*LD* longissimus-dorsi, *ST* semitendinosus, *CSA* cross-sectional area

^*^p < 0.05, **p < 0.01

## Discussion

This study elucidates the differential expression patterns of Myf5 and Myf6 genes among weaned kids of Turkish indigenous goat breeds, which may significantly influence muscle fiber composition and growth trajectories. The observed variations in gene expression align with findings from our previous research (Sen et al. 2021a), suggesting that these myogenic regulatory factors contribute to the distinct growth rates of these breeds. Notably, Honamlı kids exhibited a superior growth rate compared to Angora, Hair, and Kilis breeds, emphasizing the genetic basis of phenotypic differences in muscle mass development and fattening performance.

The genetic background underlying the differences in the phenotype of muscle mass development and fattening performance traits, especially in indigenous breeds, has yet to be fully discovered and evaluated. Moreover, Angora goats are a slower-growing breed, whereas kids of Hair and Kilis goats show relatively higher growth rates than Angora kids (Sen et al. 2021a). Our previous studies determined differences in production traits, such as growth and carcass traits (Sen et al. 2021a), and muscle fiber phenotype traits (Sirin 2018), such as fiber type, fiber numbers, and fiber size, in weaned kids born to four examined goat breeds. Additionally, prior studies have established that Honamlı and Hair kids possess a greater number of muscle fibers, particularly type II fibers, in the L and ST muscles, contrasting with the lower fiber counts observed in Angora and Kilis kids (Sirin 2018). Such disparities may stem from genomic processes governing the proliferation and differentiation of muscle precursor cells (Siqin et al. 2017), reinforcing the notion that Myf5 and Myf6 expression patterns are pivotal for muscle fiber development.

Total RNA concentration is an essential marker of muscle fibers’ transcriptional and translational efficiency and capacity (Figueiredo 2019). Previous studies have reported that small cells of the same type produce less RNA than larger ones, that the size of the cell correlates with its transcriptional activity, and that genetically induced gene expression patterns can regulate cell size (Schmidt and Schibler 1995). The current findings mirror those of previous studies (Sen et al. 2021b; Moretti et al. 2014), highlighting the relationship between RNA levels and myogenic gene expression during muscle development. Therefore, differences in RNA concentrations in some skeletal muscle masses among kids born from Turkish native breeds may be due to differences in the expression pattern of myogenic genes related to mutually interacting processes during muscle development (Sen et al. 2021b). Thus, myogenic genes’ expression levels may affect muscle fibers’ composition and size in kids born from Turkish native breeds. Siqin et al. (2017) reported that myogenic regulatory factor gene expression patterns were associated with changes in muscle fiber-type composition and muscle fiber diameter of some skeletal muscle.

Our results indicate that postnatal expression of Myf5 and Myf6 varies significantly among the examined breeds, corroborating previous observations by Huang et al. (2016). Also, they found feeding regime influence Myf5 and Myf6 genes expression of a local goat breed (Xiangdong black) in southern China. Also, Pierzchała et al. (2011) reported that expression of Myf5 in porcine skeletal muscle did not differ significantly between pig breeds. In contrast, they found that Myf6 expressed significant differences at the transcriptional level of some skeletal muscles between pig breeds. Likewise, Ropka-Molik et al. (2010) reported essential differences between pig breeds’ Myf6 expression levels of some skeletal muscles. Similarly, our findings indicated that gene expression patterns between examined four goat breeds showed differences consistent with previous studies (Ropka-Molik et al. 2010; Pierzchała et al. 2011). Moreover, results of current study may also suggest that the Honamlı breed’s higher Myf5 and Myf6 gene expression may be associated with higher muscularity of post-fattening carcasses.

Previous studies indicated that the MRFs gene family polymorphisms might be required for myotube fusion, maturation, and maintenance of skeletal muscle weight (Wyszynska-Koko et al. 2006). Moreover, previous studies determined that Myf5 and Myf6 genes polymorphisms correlated with growth traits and carcass weight in cattle (Li et al. 2004; Wang et al. 2011), pig (Wyszynska-koko and Kuryl 2004) and sheep (Siqin et al. 2017). Interestingly, several studies on Myf5 and Myf6 have shown that their polymorphisms do not affect the expression level of these genes (Urbanski and Kuryl 2004; Wyszynska-Koko et al. 2006). Therefore, the breed-specific differences in the expression level of Myf5 and Myf6 genes observed in the current study may also represent indirect effects of another myogenic regulatory mechanism expressed in goat skeletal muscle. However, the presence of some regulatory elements that may affect the expression profile of Myf5 and Myf6 indicates a very complex pattern of regulation of these genes, suggesting that there may be significant differences between breeds (Maak et al. 2006). In addition, some studies indicate that Myf5 and Myf6 genes may be expressed at low levels in myofibers (Londhe and Davie 2011). Therefore, the variation observed in the breed-dependent expression of Myf5 and Myf6 genes in goat skeletal muscles in the present study may represent the commonality of transcriptional activity of satellite cells and myofibers.

Expression patterns of MRFs gene exhibit differences in some skeletal muscles due to differences in fiber types (Siqin et al. 2017), nutrition (Vestergaard et al. 2000; Huang et al. 2016), and exercise activity (Reimers et al. 2014) in animals. However, the results of the current study show that alterations in fiber type number and MRF expression levels may have composited effects on muscle hypertrophy in kids from examined four goat breeds during postnatal development. Skeletal muscle development is a highly coordinated process involving a unique signal regulatory mechanism. Various elements, such as some genes, miRNAs, and lncRNAs, were determined to participate in fetal and postnatal muscle growth and development through a lot of signaling pathways. MRFs gene family, paired box protein 3/7 (Pax3/7), Myostatin (MSTN), and myocyte enhancing factor 2 (MEF2) family can be given as examples of the fundamental factors that regulate the growth of muscles (Huang et al. 2016). Although MRFs family genes control the skeletal muscle cells differentiation during fetal and postnatal myogenesis, postnatal expression of these genes can be significantly affected by nutrition and exercise intensity (Vestergaard et al. 2000; Siqin et al. 2017). In the current study, although all kids were raised under similar environmental conditions, significant differences were found in the expression of Myf5 and Myf6 among kids born to four Turkish indigenous goat breeds. The expression patterns of the Myf5 and Myf 6 genes were relatively critical, especially in the LD skeletal muscle of Honamlı kids, suggesting that kids have more muscle mass and live weight by improving the development of muscle fibers. This situation was consistent with the fact that Honamlı kids have more outstanding total edible meat content and heavier carcass parts in their carcasses. This result also revealed low carcass weight and total edible meat content in Angora and Kilis kids, which showed low expression patterns of Myf5 and Myf 6 genes in LD and ST skeletal muscles. qRT-PCR analysis of muscle tissue indicated higher expression levels of Myf5 and Myf6 genes in Honamlı kids’ LD muscle. This finding suggests a potential genetic basis for the observed weight differences, with specific genes associated with muscle development showing higher expression levels in the Honamlı kids.

To our knowledge, this is the first study to examine the breed-related relationships of Myf5 and Myf6 genes expressed during the postnatal skeletal muscle growth period, as well as to show a relationship between muscle fiber type composition and size and Myf5 and Myf6 expression patterns in different skeletal muscles of weaned kids born to Angora, Hair, Honalı and Kilis Turkish indigenous goat breeds. This study presented significantly different expressions of Myf5 and Myf6 genes in investigated skeletal muscles among weaned kids from Turkish indigenous goat breeds. Although many reports have addressed how different exercises or stimuli affect myogenic regulatory factors, including Myf5 and Myf6 gene expression, muscle fiber conversion, and compositions (Reimers et al. 2014; Huang et al. 2016; Siqin et al. 2017), the specific molecular mechanisms governing muscle development still need clarification. Significant differences between Myf5 and Myf6 gene expressions may allow us to select both candidate genes for further meat production trait-associated studies. Additionally, since many different molecular mechanisms can regulate the expression activity of specific genes that regulate the growth and development of muscle fibers (Pierzchała et al., 201; Siqin et al. 2017), further defining the causal polymorphism and determining their functional roles is necessary.

In Angora kids, the expression of the Myf6 gene exhibited intriguing correlations with muscle fiber characteristics in the ST muscle. Similarly, although no significant correlation was observed between gene expression of Myf5 and fiber properties of LD muscle, Myf6 gene expression showed substantial correlations with fiber properties in Hair Kids. In Honamlı kids, Myf6 gene expression correlated with the number of fiber types in the ST muscle, suggesting its involvement in regulating fiber number in this breed. Interestingly, the expression of Myf5 and MYF6 genes in Kilis kids showed varying correlations with fiber type composition in LD and ST muscles, which may suggest a complex regulatory role of the MRFs gene family in fiber type distribution and size in different muscles in this breed. Overall, these results highlight the intricate relationship between Myf5 and Myf6 gene expression and muscle fiber characteristics across different breeds and muscles.

The observed correlations between Myf5 and Myf6 expression and muscle fiber characteristics, particularly in the ST muscle of Angora and Hair kids, highlight the intricate regulatory roles these genes play in fiber type distribution and size. Overall, the findings emphasize that breed-specific gene expression patterns can be leveraged to select for more productive goats, particularly in fattening contexts. Results of the current study indicated that alterations in muscle fiber number, type, and size might be associated with interactive activity of Myf 5 and Myf 6 gene expression during postnatal development. Moreover, significantly different breed-specific expressions of Myf5 and Myf6 led to the conclusion that these genes can be used to choose more productive goat breeds, especially in fattening under the same conditions. Moreover, this study contributes valuable information to understanding the complex interplay between breed and genetic factors in growing animals.

In conclusion, this study contributes to the understanding of the genetic factors influencing muscle development in Turkish indigenous goat breeds. The differential expression of Myf5 and Myf6 offers valuable insights for breeding strategies aimed at optimizing growth and meat quality. Future research should focus on elucidating the genetic mechanisms underpinning these differences, which will enhance our capacity to improve livestock performance through targeted breeding and management practices.

## Data Availability

The datasets generated during and/or analyzed during the current study are not publicly available due to some authors’ concerns but are available from the corresponding author on reasonable request.
